# Medical Society of the County of Erie

**Published:** 1899-07

**Authors:** F. C. Gram


### MEDICAL SOCIETY OF THE COUNTY OF ERIE.

### SEMI-ANNUAL MEETING.

REPORTED BY F. C. GRAM, M. D., Secretary.

THE Medical Society of the County of Erie held its semi-annual meeting, June 13, 1899, at 10 a. m., in the parlors of the Buffalo Academy of Medicine.

The president, Dr. J. B. Coakley, took the chair at the appointed hour. The minutes of the annual meeting were read and approved.

Dr. William Warren Potter, chairman of the Committee on membership, presented the names of the following applicants as having qualified in accordance with the by-laws, and moved that they be admitted to the society: H. B. Brownell, Edward J. Kiepe, William B. May, Nelson W. Wilson, Montressor Axford, H. R. Gaylord, Charles A. Brownell and Charles F. Durand.

The report was adopted and the applicants elected to membership.

Applications for membership were received from Dr. W. J. Dean, James W. Nash and Ernest N. Ewell.

Referred to the committee on membership. The vice-president, Dr. E. H. Ballou was called to the chair and Dr. Coakley read the semi-annual report of the Board of Censors as follows:

*To the Medical Society of the County of Erie:*

The board of censors respectfully reports: That during the year since the last annual meeting of this society, it has endeavored to perform its duties in discovering and bringing to punishment all persons who unlawfully practice medicine or surgery in this county. All complaints concerning such practices have been carefully investigated, not only by the members of this board but also in some instances by detectives employed for that purpose, under the advice of our legal counsel, Hon. Tracy C. Becker. In one case of a flagrant character, that of Stephen T. Lee, who has for some years been administering medicine to many persons in this city for hire for cancer and other diseases, without being duly licensed as a physician, we believe that we have obtained sufficient evidence and a complaint has recently been presented to the police justice, and the defendant arrested. His case will be heard today (June 13th) before the Police Court. It is believed that said Lee, whose ordinary occupation is that of day laborer on the docks, has accumulated property of a considerable amount in value by his illegal practice, and if warranted by the evidence we have secured, and we are so advised by our counsel, we shall, besides prosecuting him criminally, institute a suit against him in the Supreme Court, to recover numerous penalties of $250 for each offense under the provisions of the law of 1896.

### ROLFE SAUNDERS'S DEATH.

Within the past few weeks a case of more than ordinary importance has come before the United States Court, growing out of the death of a child named Rolfe Saunders, at Fort Porter, in this city, who, it is claimed, died of double pneumonia, under the care of one George H. Kinter, a so-called christian scientist healer, who had been called in to cure this child by its father, James C. Saunders. The post mortem examiners' report of the autopsy performed by them in this case indicated so clearly that the disease had existed for some days, and could have been discovered by the usual methods of examination and diagnosis if the child had been attended by a duly licensed physician of ordinary skill, that the District Attorney, Hon. Emery P. Close, deemed it his duty to cause the arrest of the father and mother of the child and of the alleged healer Kinter, on the charge of manslaughter; the United States authorities and not the state authorities, having exclusive jurisdiction of this charge, because the death occurred on United States Government Reservation. After a considerable amount of testimony had been given on these charges before Hon. Charles E. Robinson, United States Commissioner, this board, after consulting many members of this society, deemed it advisable that our counsel should be invited to assist the United States District Attorney in prosecuting the medico-legal aspects of the matter.

### ASSISTED MR. CLOSE.

The United States Attorney cheerfully and courteously consented that this should be done, and our counsel has given him valuable assistance in prosecuting the case, which on Monday, June 12th, resulted in the healer Kinter and the father Saunders being held by the United States Commissioners in $2,000 bail each, to answer the charge of manslaughter at the next term of the United States District Court of the Northern District of New York, which will be held in this city in September next.

It would not be proper for us to discuss the facts disclosed by the evidence in this matter, but in the opinion of your board the charges against this man should be prosecuted vigorously and to the full extent of the law.

### TRACY C. BECKER TO ASSIST.

While the zeal and ability of the United States District Attorney are fully recognised, the medico-legal features of this case are such that we feel sure that he will welcome in the futuie, as he has in the past, the aid of our counsel, who has long made special study of these subjects. We, therefore, suggest that this society should adopt a resolution requesting the United States Government to assign our counsel, Tracy C. Becker, as special counsel to assist in prosecuting this case in order that he may have official standing and recognition before the United States District Court.

We further report that, following the course pursued by the Medical Society of New York County, we have, with the assistance of our counsel, caused a list of all the duly licensed and registered physicians appearing on the records of the county's clerk's office to be prepared and compared with the names of the physicians of this city as they appear in the Buffalo City Directory for 1898.

### PHYSICIANS MUST REGISTER.

We have also prepared and sent by mail to sixty-six persons whose names appear in the Directory of 1898 as physicians, but do not appear on the records of the county clerk's office to be duly registered as such, a circular letter, a copy of which we attach to the report. To this circular letter we have received about thirty replies. We are still engaged in pursuing our investigations along these lines, and when the new city directory is issued, the latter part of this month, it may be necessary to send out new circular letters to other persons who by accident or design have not been registered as required by law. We respectfully ask that our action in all these matters be approved, and that the same amount of money as was allowed last year be appropriated this year for the purpose of compensating our counsel and of defraying the necessary disbursements to be received by the new board of censors in the performance of its duties.

COPY OF CIRCULAR.

*Dear Sir*—The Board of Censors of the Erie County Medical Society, by direction of that society, has been endeavoring to suppress the illegal practice of medicine in this county. It is the duty and intention of the board to prosecute all persons who are practising medicine and surgery who have not been duly licensed and registered, as required by Chapter 661 of the Laws of 1893, and the other statutes of this state. All lawful practitioners will approve this action and assist us by all means in their power. The board has caused a list to be prepared of all persons practising in this county, and particularly in the city of Buffalo, who have been duly registered in the county clerk's office, as required by law. On comparing this list with the directory of the city of Buffalo we find that your name appears in the directory as a physician, while it does not appear on the records of the county clerk's office as being duly registered there.

Will you kindly call upon, or otherwise inform the undersigned chairman of the board, whether this is correct or not, and if by mistake or inadvertence your license to practise is not registered, you should have it registered at once and notify the undersigned that you have registered it? Yours, etc.

Respectfully submitted,

J. B. COAKLEY, *Chairman.*
THOS. F. DWYER,
IRVING W. POTTER,
CHAS. E. CONGDON,
ALBERT FRONCZAK.

On motion the report was received and filed.

Dr. WILLIAM WARREN POTTER presented the following resolution, which was adopted :

*Resolved*, That the secretary of the Medical Society of the County of Erie be, and he is hereby directed to address the proper officer of the general government requesting that, if agreeable to the practice, an invitation be extended to the Hon. Tracy C. Becker, counsel for this society, to attend at any further proceeding, hearing or trial relating to the recently deceased child, Rolfe Saunders, for the causing of, or contributing to, which George H. Kinter, a so-called christian scientist, has been held in bail to appear before the United States grand jury.

On motion of Dr. Eli H. Long the treasurer was empowered to disburse a reasonable sum of money for the employment of detectives to assist in the prosecution of illegal practitioners.

Dr. William Warren Potter stated that the treasurer, Dr. Edward Clark, had prepared a list of members who are in arrears of dues for three years or more. He moved that the treasurer read the list and that they be suspended from the privileges of membership until payment, in accordance with the by-laws.

The motion was unanimously adopted and the treasurer read the names as coming within this provision.

Dr. Eli H. Long called attention to the fact that several members of the society advertise their practice in violation of the by-laws.

Dr. Coakley asked Dr. Long to submit the cases in writing and the Board of Censors would take cognisance.

Papers were read as follows: Eclampsia infantum, Dr. Thomas F. Dwyer; A study of the pathogenesis of gout, Dr. Mary Clayton; The importance of early diagnosis and surgical intervention in fractures of the skull, Dr. Marshall Clinton; Malaria complicating the puerperium, Dr. G. A. Himmelsbach; A protest, Dr. J. J. Finnerty; Prophylaxis in obstetrics, Dr. L. Schroeter. (These papers are to appear in a future number of the Journal.)

Discussion followed the reading of these papers, and after passing a vote of thanks to the contributors the society adjourned.

---

### SPECIAL MEETING.—ERIE COUNTY MEDICAL SOCIETY.

Reported by F. C. GRAM, M. D., Secretary,

The Medical Society of the County of Erie was called together at 5 p. m., June 20, 1899, by Dr. J. B. Coakley, president, who announced the death of Dr. W. C. Earl, and asked the society to take appropriate action.

On motion a committee consisting of Drs. Sage, Wyckoff and Hopkins was appointed to draft a suitable memorial. The committee presented the following:

Forasmuch as it hath pleased Almighty God, in His wise providence, to take out of this world the soul of Wesley Clark Earl, M. D., for a quarter of a century an honorable and honored member of the Medical Society of the County of Erie, we therefore bear willing testimony and make record of our hearty appreciation of his many excellent qualities of mind and person, of his modest, upright and worthy professional career, of his high character as a member of the family, of the state and of the profession of medicine, to which he gave his life; therefore

*Resolved*, That we place upon our records and transmit to the family of the deceased this tribute and testimony; and

*Resolved*, That we tender to the bereaved family our sincere sympathy and condolences, and that we attend the funeral.

C. A. SAGE,
C. C. WYCKOFF,
H. R. HOPKINS.

Remarks were made by members present. Dr. D. W. Harrington was intimately acquainted with Dr. Earl during his entire practice. Dr. Grosvenor said he was a former pupil of the deceased, and knew him as a tutor, medical student and practitioner. He was conscientious professionally, socially and in business. Dr. T. M. Johnson, likewise an old friend, spoke of the deceased's good qualities. Dr. Sage said he was the first medical student whom Dr. Earl instructed. He was a good physician and surgeon and respected his calling with honor. Dr. Hopkins said that owing to the quiet and retired manner of the deceased his true worth did not always become apparent. He also spoke of his final illness. Dr. Wyckoff said that Dr. Earl always exemplified the Golden Rule.

The memorial was then adopted and the society adjourned.

